# Innovative Infrared Spectroscopic Technologies for the Prediction of Deoxynivalenol in Wheat

**DOI:** 10.1021/acsfoodscitech.4c00730

**Published:** 2025-01-08

**Authors:** Polina Fomina, Antoni Femenias, Miriam Aledda, Valeria Tafintseva, Stephan Freitag, Michael Sulyok, Achim Kohler, Rudolf Krska, Boris Mizaikoff

**Affiliations:** 1 Institute of Analytical and Bioanalytical Chemistry, Ulm University, Albert-Einstein-Allee 11, Ulm 89075, Germany; 2 Faculty of Science and Technology, Norwegian University of Life Sciences, Dro̷bakveien 31, Ås 1432, Norway; 3 Department of Agrobiotechnology IFA-Tulln, Institute of Bioanalytics and Agro-Metabolomics, University of Natural Resources and Life Sciences, Vienna, Konrad Lorenzstr. 20, Tulln A-3430, Austria; 4 Institute for Global Food Security, School of Biological Sciences, Queen’s University Belfast, 19 Chlorine Gardens, Belfast BT9 5DL, Northern Ireland; 5 Hahn-Schickard, Sedanstraße 14, Ulm 89077, Germany

**Keywords:** ATR, QCL, IR, mycotoxins, deoxynivalenol, SPLS-DA

## Abstract

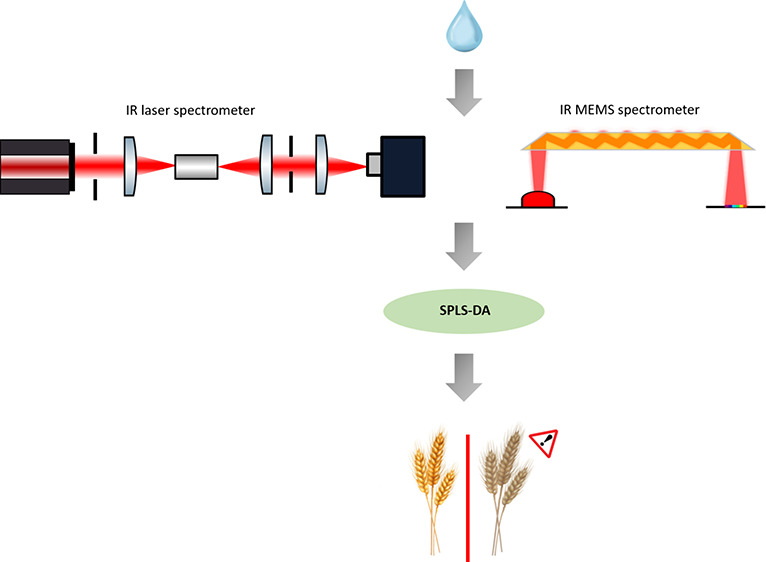

Mycotoxin contamination in cereals is a global food safety concern. One of the most common mycotoxins in grains is deoxynivalenol (DON), a secondary metabolite produced by the fungi*Fusarium graminearum* and *Fusarium culmorum*. Exposure to DON can lead to adverse health effects in both humans and animals including vomiting, dizziness, and fever. Hence, the development of analytical technologies capable of predicting mycotoxin contamination levels in grains is crucial. In this study, we emphasize innovative infrared (IR) spectroscopic technologies for the prediction of DON in wheat along the food supply chain. The performance of an IR laser spectroscopic platform for on-site or laboratory confirmative analysis was evaluated. Furthermore, the performance of a handheld IR spectrometer for preliminary screening during transportation, storage, or harvesting was assessed. The accuracy of cross validation (Acc_CV_) obtained with the laser spectrometer reached 92%, while the handheld IR spectrometer achieved 84.6%. Hence, both technologies prove significant potential for rapid mycotoxin detection.

## 1 Introduction

Mycotoxins are toxic secondary metabolites produced by certain filamentous fungi. The most prevalent species in the moderate climatic regions of the world are *Fusarium* species such as *F. graminearum* and *F. culmorum*. One of the most frequently occurring *Fusarium* mycotoxins in grains is deoxynivalenol (DON) although evidence of adverse health effects in humans due to chronic exposure to DON is still lacking. Human intoxication from acute exposure to DON has been repeatedly reported in Asia with symptoms including nausea, vomiting, diarrhea, abdominal pain, headaches, dizziness, fever, and in severe cases bloody stool.^1^ The effects of DON on the immune response of farm animals lead to an increased susceptibility to infectious diseases at medium to high doses of DON (8–10 mg/kg bw). Oral exposure to DON leads to both developmental and reproductive toxicity in animals including reduced fertility, embryotoxicity, skeletal abnormalities, effects on body weight, and postnatal mortality.^1^ Hence, the presence of DON in grains and cereal-based products is a global food and feed safety issue. As a result, there is a demand in food industries and among farmers for technologies that enable on-site rapid screening of DON and other mycotoxins in grains.^2−4^

Routine confirmatory methods for mycotoxin analysis include liquid chromatography coupled to mass spectrometry (LC-MS/MS)^5^ and screening methods such as enzyme-linked immunosorbent assay (ELISA) or lateral flow devices (LFD).^6^ While being considered gold-standard methods, limitations of LC-MS/MS for mycotoxin analysis include cost and the requirement of a laboratory environment, while ELISA and LFD suffer from cross-reactivity and require a notable number of consumables.^7,8^

In the recent years, vibrational spectroscopic methods, particularly mid-infrared (MIR) spectroscopy, have gained increasing acknowledgment in food safety scenarios.^9^ Taking advantage of molecular vibrational transitions, specific vibrational modes are selectively probed, offering detailed information on the chemical composition and molecular structure of the sample. The nondestructive and rapid nature of MIR spectroscopic techniques addresses some of the challenges in routine food analysis as only minimal sample preparation is needed, devices can be portable, a laboratory environment is not necessary, and consumables are minimized, which facilitates on-site and in-field analysis.^10^

MIR spectroscopy combined with chemometrics has been previously utilized to predict levels of different types of mycotoxin contaminations in various commodities via changes in the MIR absorption spectrum induced by fungal infections. For instance, Femenias et al. discriminated between high and low DON contaminated corn according to the European Union (EU) regulatory level (1750 μg/kg), applying a classification method based on sparse partial squares discriminant analysis (SPLS-DA). The classification accuracies of predictions were 86.7% and 90.8% depending on the extraction solvent.^11^ In another study, Galvis-Sanchez et al. could separate dried fruits contaminated with ochratoxin A from noncontaminated ones by investigating scatter-plots and loadings using principle component analysis (PCA).^12^ In addition, studies using MIR spectroscopy for mycotoxin prediction in other various commodities have been reported.^9,13,14^ These reports use conventional Fourier transform mid-infrared (FTIR) spectroscopic systems operating in the 2.5–25 μm regime typically sampling via attenuated total reflection (ATR).^15^

In the present study, it is demonstrated that food analysis via IR spectroscopy is not limited to conventional FTIR approaches but goes beyond the current state-of-the-art by utilizing innovative IR technologies. This includes, for example, a handheld IR microelectromechanical system (MEMS) device for rapid on-site screening of mycotoxins along the food supply chain, e.g., during transportation, storage or harvesting, and a laser-based IR spectroscopy for sophisticated confirmatory analysis on-site or in a laboratory.

The motivation for this study is driven by a stakeholder survey conducted by the consortium of the EU Horizon Project PHOTONFOOD.^2^ In this survey, organizations across the entire food supply chain including 22 analytical laboratories, 24 food processing companies, 21 agricultural companies, and institutions in food trade and food control have participated. The stakeholders expressed partial dissatisfaction with current mycotoxin monitoring methods citing high analysis costs, extended measurement times, and the requirement for trained personnel to operate specific equipment. The readiness to adopt novel technologies, if they could provide a low-cost, rapid prescreening tool for farmers and food processors or a sensitive, fast-acting device for regulatory authorities and service laboratories, was clearly present. Therefore, this study aims at addressing stakeholders’ demands by introducing innovative IR methodologies and techniques.

The laser-based device uses broadly tunable external cavity quantum cascade lasers (EC-QCLs), which avoid the need for an interferometer while using a single-element detector. Nowadays, up to four EC-QCLs are implemented providing a combined spectral coverage of the relevant fingerprint range.^16−18^ Given the high brightness, laser spectroscopy is particularly suited for chemical sensing in highly absorbing samples or if high sensitivity is required.^19,20^ Compared to a conventional FTIR laboratory spectrometer, QCL-based systems provide significantly lower noise levels, and accordingly, improved signal-to-noise ratios (SNR). For example, proteins were determined at concentrations <0.1 g/L with a limit of detection (LOD) of 0.0043 g/L using a QCL spectrometer with balanced detection, while the corresponding LOD by an FTIR spectrometer was higher at 0.035 g/L.^21−23^

To date, only a few examples have been reported using laser-based technologies for mycotoxin screening. Sieger et al. could separate aflatoxin B_1_ contaminated vs noncontaminated peanut samples as well as maize contaminated with DON according to the EU regulatory limits.^24^ It was shown that moldy peanuts can be distinguished from healthy ones utilizing IR laser spectroscopy.^25^ Given the potential of this technology, it was investigated whether this technology may be adopted for predicting DON levels in wheat and to assess whether conventional FTIR spectroscopy is outperformed meeting the stakeholder demands for sensitive and rapid analytical tools.

A less sensitive yet unprecedentedly compact technology is IR spectroscopic sensing platforms utilizing MEMS light sources emitting a broad spectral range coupled to an ATR sampling waveguide. Again, to avoid an interferometer, a pyroelectric line array detector (PLAD) combined with a linear variable filter (LVF) provides the required wavelength discrimination. This combination uniquely enables hand-sized portable MIR devices without any moving parts, which addresses the limitation of FTIR spectrometers, which are rather difficult to take into the field; likewise, the weight and dimensions of FTIR spectrometers limit their portability vs the developed handheld device at a mere 0.5 kg.^26^ An appropriate selection of the LVF enables tailoring of the system for the MIR spectral window of interest. The performance of this technology was previously evaluated using model scenarios including DON detection achieving analytical figures-of-merit similar to or only slightly inferior to conventional FTIR laboratory systems.^27^ Therefore, on-site rapid screening of mycotoxin contamination in the field, during transportation, or in storage scenarios appears feasible.

In the present study, these technologies were employed to classify wheat samples with high and low levels of DON contamination according to the EU regulatory limit (i.e., for wheat 1250 μg/kg),^28^ demonstrating their application abilities along the food supply chain.

## 2 Material and Methods

### 2.1 Sample Preparation

Lantmännen SW Seed AB (Switzerland) and KWS Lochow GmbH (Germany) provided two different wheat varieties (Kronjet and Lennox) that were planted and harvested at different seasons. Wheat with the minimum DON contamination was guaranteed by applying Folicur (1L/ha), as specified in the standard agricultural practices. Alternatively, DON-contaminated samples were obtained by inoculation via kernel-pawn and spraying methods of *F. graminearum* and *F. culmorum*. Infected maize kernels by *F. graminearum* were allocated on the soil (15 g/m^2^) 21 days before flowering, ejecting ascospores to the air and promoting wheat ears infection. On the other hand, the spraying of a 50.000 conidia/mL solution of *F. graminearum* and *F. culmorum* (100 mL/m^2^) during anthesis ensured the infection of wheat in field. Contaminated and noncontaminated wheat batches were blended. For that purpose, the two fractions were located in a plastic bag, which was inflated with air and shaken by hand for 10 min. After the samples were mixed, the kernels were finely ground using a laboratory mill (Romer, Union, MO, USA). Several DON contamination levels of the Kronjet variety were prepared by blending a sample containing 6020 μg/kg of DON with a blank sample, thereby obtaining subsamples with 0 (below the LOD of the reference method), 100, 200, 500, 750, 1250, 1750, 2000, 3000, 4000, 5000, and 6020 μg/kg DON. Likewise, Lennox wheat blending consisted of a sample with a DON concentration of 10600 μg/kg of DON mixed with a blank sample resulting in subsamples with 0, 100, 200, 500, 750, 1250, 1750, 2000, 3000, 4000, 5000, 6000, 7000, and 10600 μg/kg of DON. As a result, 26 wheat samples were prepared. The whole wheat flour was stored at 4 °C until extraction. Before being subject to analysis via IR spectroscopy, DON concentrations were quantified by a validated reference method (LC-MS/MS) using a QTrap 550 System (Applied Biosystems, USA), which was described in detail in studies by Fomina et.al. and Sulyok et al.^29,30^ The summarized information on the sample set is represented in Table 1.

**Table 1:** Statistical Summary of the DON Content in the Analyzed Sample Set

statistical parameter	sample set
no. of samples	26
concentration range, μg/kg	0^a^–10600
mean concentration, μg/kg	2780
median concentration, μg/kg	1875
threshold, μg/kg	1250

a Below the LOD of the reference method.

For extraction, two different water-based solvents (v/v, shown in parentheses) were used: Milli-Q water (100) produced by a Thermo Scientific Barnstead NanoPure System (Waltham, Massachusetts, U.S.) and an ethanol:water (30:70) mixture with ethanol 99.5% (VWR chemicals, Radnor, Pennsylvania, U.S.). The extraction procedure was the same as reported by Fomina et.al.^29^ Briefly, 2 g of the homogenized wheat material was mixed with 8 mL of solvent in a 15 mL tube and facilitating the solid–liquid transfer by shaking for 60 min at 120 rpm on a Rocking platform (VWK, UK). The mixtures were centrifuged for 2 min at 6000 rpm, and 2 mL of the supernatant was transferred to an Eppendorf tube (Eppendorf AG, Hamburg, Germany). Samples were stored at 4 °C until spectroscopic analysis.

### 2.2 IR Laser Spectrometer

**Figure 1:**
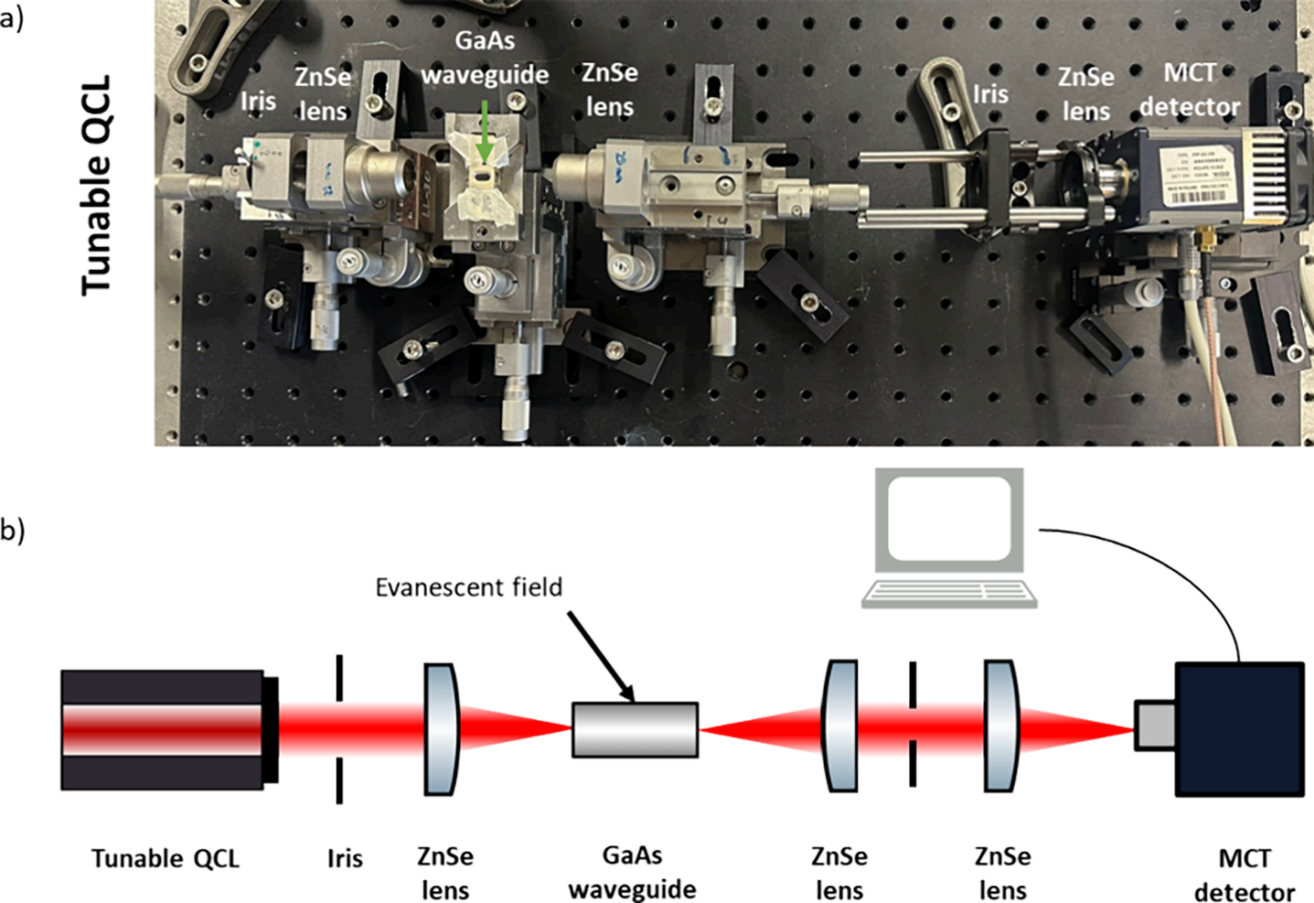
(a) IR laser spectrometer and (b) schematic of the beam path within the IR laser spectrometer. (QCL = quantum cascade laser, ZnSe = zinc selenide, GaAs = gallium arsenide, MCT = mercury cadmium telluride).

The IR laser spectrometer comprises a broadly tunable EC-QCL system (MIRcat-QT, Daylight Solutions, USA) emitting IR radiation covering the spectral range 1850–1550 cm^–1^ operated in pulsed mode. IR radiation emitted by the laser is focused via 1/2″ planoconvex ZnSe lenses (Thorlabs, Germany) on a thin-film GaAs waveguide chip (5 × 10 mm) (Figure 1a) with a waveguide layer thickness of 6 μm serving as a miniaturized evanescent field sensing element. Details on the waveguide technology pioneered by the team of Mizaikoff can be found in the other publications of the research group.^31−33^ Radiation emanating at the distal end of the waveguide is collimated and focused on a thermoelectrically cooled mercury–cadmium-telluride (MCT) detector (Vigo, Poland; Figure 1a). For a schematic of the beam path, see Figure 1b.

An aliquot of 10 μL of sample extract was deposited at the waveguide surface. All samples were dissolved in water (100) or ethanol:water (30:70). Spectra of samples were recorded after evaporation of the solvent, reconstituting an analyte film at the waveguide surface under air flow. The evaporation times were 8 and 15 min for ethanolic and aqueous extracts, respectively. IR spectra were acquired within approximately 5 min at a spectral resolution of 2 cm^–1^. Each sample was measured in three technical replicates that were averaged at the data analysis step.

### 2.3 IR MEMS Spectrometer

**Figure 2:**
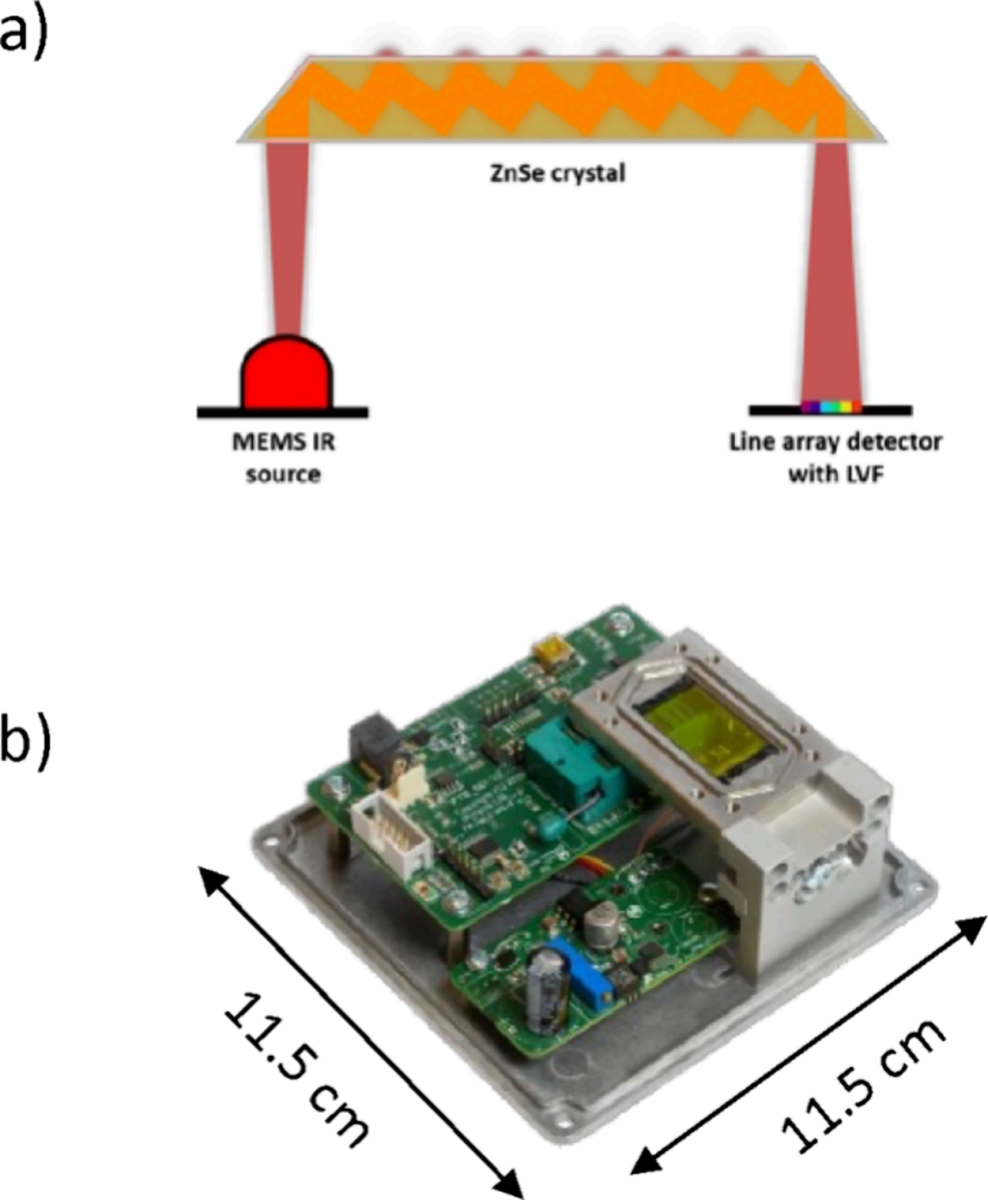
(a) Schematic of the beam path within the IR MEMS spectrometer; (b) IR MEMS spectrometer. (IR – infrared; MEMS = microelectromechanical system; ZnSe = zinc selenide, LVF = linear variable filter).

The IR MEMS spectrometer consists of a MEMS-based IR light source, a multireflection ZnSe ATR waveguide (50 × 20 × 2 mm), and a PLAD detector with LVF for wavelength discrimination (form Broadcom, USA (formerly Pyreos Ltd., UK)). Each PLAD comprises 128 individual sensor elements (i.e., pixels). The LVF was optimized for the spectral range of approximately 1818–909 cm^–1^ (5.5–11.0 μm). The schematic in Figure 2a illustrates the beam path. The dimensions of an IR MEMS spectrometer along with all required electronic are 11.5 × 11.5 × 4.5 cm (width × depth × height) and the weight is 500 g (Figure 2b).

For each measurement, 400 scans were averaged (background and sample). To cover the entire sensing area of the ATR waveguide, 0.5 mL aliquots of the sample extracts were deposited. The spectra were recorded after solvent evaporation under an air flow. The evaporation times were 7 and 40 min for ethanolic and aqueous extracts, respectively. IR spectra were acquired within approximately 2 min. Each sample was measured in three technical replicates, which were averaged at the data analysis step.

### 2.4 Data Analysis

The IR spectra obtained from both devices were subjected to preprocessing before data analysis. For IR laser data, the following preprocessing steps were applied: (1) smoothing with the Savitzky–Golay algorithm with a window size of 13 and a second polynomial order; (2) normalization by extended multiplicative scatter correction (EMSC) with linear and quadratic terms. For the IR MEMS data, preprocessing procedures were applied as follows: (1) smoothing with the Savitzky–Golay algorithm with a window size of 7 (ethanol:water (30:70)) or 21 (water (100)) and a second polynomial order; (2) normalization by extended multiplicative scatter correction (EMSC) with linear and quadratic terms.^34^

SPLS-DA was carried out to obtain classification models. The method was selected due to its effectiveness as a robust multivariate dimensionality reduction tool combined with superior interpretability compared to other multivariate approaches such as Artificial Neural Networks (ANN) or Support Vector Machines (SVM). SPLS-DA provides regression coefficients, enabling the identification of wavelengths that contribute most significantly to the predictive models. This facilitates the correlation of spectral variations with specific wheat components, such as proteins, carbohydrates, fats, etc. across different solvents. SPLS-DA with 99% sparsity was applied to focus on the most important spectral variables^35,36^ i.e., for each PLS component, 99% of the variables were penalized and thus set to zero and only 1% of the variables were used to construct the PLS component. This allows the elimination of unnecessary variables and ambiguous correlations between spectral variables.

A total of 26 wheat samples were analyzed to build the models, comprising 14 highly contaminated and 12 low contaminated samples, with their respective concentrations determined via reference LC-MS/MS analysis (Section 2.1). To delineate between classes (high vs low DON contamination), a threshold of 1250 μg/kg was established in accordance with the EU regulations (COMMISSION REGULATION (EU) 2023/915 of 25 April 2023) governing DON contamination in wheat.^28^ All of the models were built using leave-one-out cross validation (CV), whereby three technical repetitions of the same sample were taken out at each step of the CV to avoid overfitting. By use of CV, the limitations associated with the training and test validation approach were mitigated. The latter would have resulted in a relatively small number of samples, thereby compromising the representativity of both the training and test sets.

Classification accuracy of CV (Acc_cv_) was used as a metric of model performance as this represents the ratio of correct predictions to the total number of predictions. Regression coefficients, the number of latent variables (LV), specificity (model ability to predict true negatives of each category), and sensitivity (model ability to predict true positives of each category) were utilized to conduct a more detailed characterization of the models. True positives in all the models are high contaminated samples, while true negative ones are low contaminated. The number of false negatives (high contaminated samples classified as low) was also provided for every model. Data analyses were performed by a combination of standard and in-house developed algorithms in Matlab, R2022a (The Mathworks Inc., Natick, Massachusetts).

## 3 Results and Discussion

### 3.1 IR Laser Spectrometer for DON Analysis

**Figure 3:**
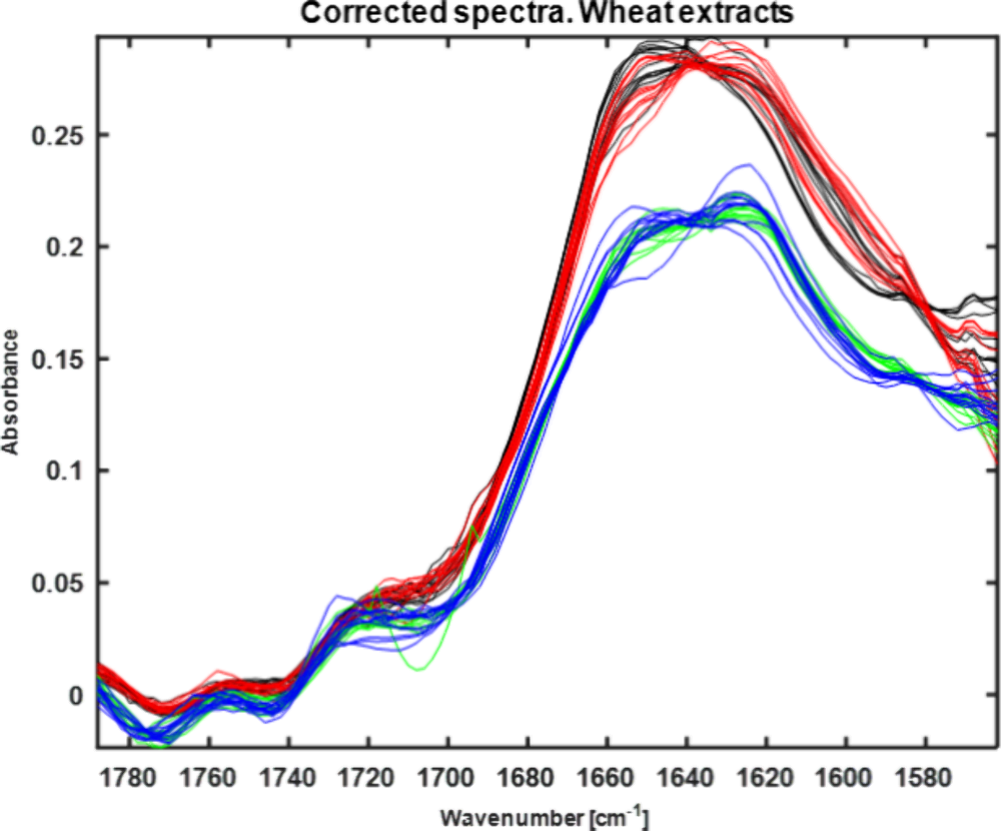
Preprocessed IR spectra of wheat extracts obtained with the IR laser spectrometer using water (100) as the extraction solvent (black line–low contaminated sample, red line–high contaminated) and using ethanol:water (30:70) (blue line–low contaminated, green line–high contaminated).

Preprocessed IR spectra obtained with the IR laser spectrometer after extraction with water (100) and ethanol:water (30:70) are represented in Figure 3. The direct identification of the carbonyl bands typical for DON (1685 cm^–1^) is not possible due to interference with bands related to additional extracted components (Table 2). Hence, changes in the IR spectra caused by DON contamination are correlated with changes in extracted matrix components generically caused by fungal growth, including DON production, which still enables the classification of wheat samples according to the DON level. From Figure 3, it is evident that the obtained spectral pattern is dependent on the extraction solvent and the DON concentration.

**Table 2:** Assignment of Wheat and DON Bands in the IR Spectra^37−40^

**wavenumber, cm**^**–1**^	**characteristic wheat bands**
1745	C=O ester stretching, lipids, carbohydrates
1642	amide I (C=O stretching): proteins, pectins
1540	amide II (C–N stretching, N–H bending): proteins
1446	C–H: cell wall polysaccharides, alcohols and carboxylic acids
1239	amide III (C–N and N–H bending): proteins

	**characteristic DON bands**
1685	C=O (carbonyl group)
1167	C–O–C antisymmetric stretching
1069	RCH–OH stretching
956	C–O (epoxide ring) stretching

The binary classification of wheat samples into low contaminated and high contaminated groups was carried out using SPLS-DA. The results are summarized in Table 3. The classification accuracy for the model using ethanol:water (30:70) as an extraction solvent is 92%, while for the water (100), it is 88%. The number of LVs in a PLS-DA model illustrates the complexity of the classification model, i.e., how many components are required to separate the groups. Table 3 indicates that the models displayed a high classification power despite low complexity, i.e., only 2 LVs for the ethanol:water extracts and 3 LVs for the water (100) extracts. Furthermore, both models classified 2 instances of high contamination as low, resulting in false negatives.

**Table 3:** SPLS-DA Classification Results of High vs. Low DON-Contaminated Wheat Extracts Obtained via the IR Laser Spectrometer^a^

**solvent**	**Acc**_**cv**_**, %**	**#LVs**	specificity, %	sensitivity, %	**no. of false-negative samples**
ethanol:water (30:70)	92%	2	100%	83%	2
water (100)	88%	3	92%	85%	2

a Acc_CV_ – accuracy of cross-validation, #LV – number of latent variables.

An analysis of the results shows that the difference in performance between water and the ethanol:water (30:70) mixture is minor (4% variation). Both solvents demonstrate Acc_cv_ greater than 87%, which, according to the literature, is considered an advanced result,^41^ ensuring an eco-friendly method due to relatively green extraction solvents. There are only a limited number of publications in which DON was determined in wheat utilizing MIR spectroscopy. For instance, in 2019, De Girolamo et al. evaluated the performance of Fourier transform mid-infrared spectroscopy in determining DON in wheat bran extracts employing PLS-DA and principal component-linear discriminant analysis (PC-LDA). Different spectra preprocessing strategies were utilized, and the best performing one (smoothing, baseline correction, and MSC) demonstrated the following results of discriminant analysis: FTIR showcases accuracies for PLS-DA and PC-LDA of 86% and 87%, respectively. Both models underperformed compared to the one achieved in the current study with IR laser spectrometer, demonstrating strong analytical capabilities for the developed device. However, by combining FTIR spectra with the spectra obtained with Fourier transform near-infrared spectroscopy (FTNIR), the authors achieved comparable accuracy to the laser spectrometer being 91% for both methods PLS-DA and PC-LDA.^42^

**Figure 4:**
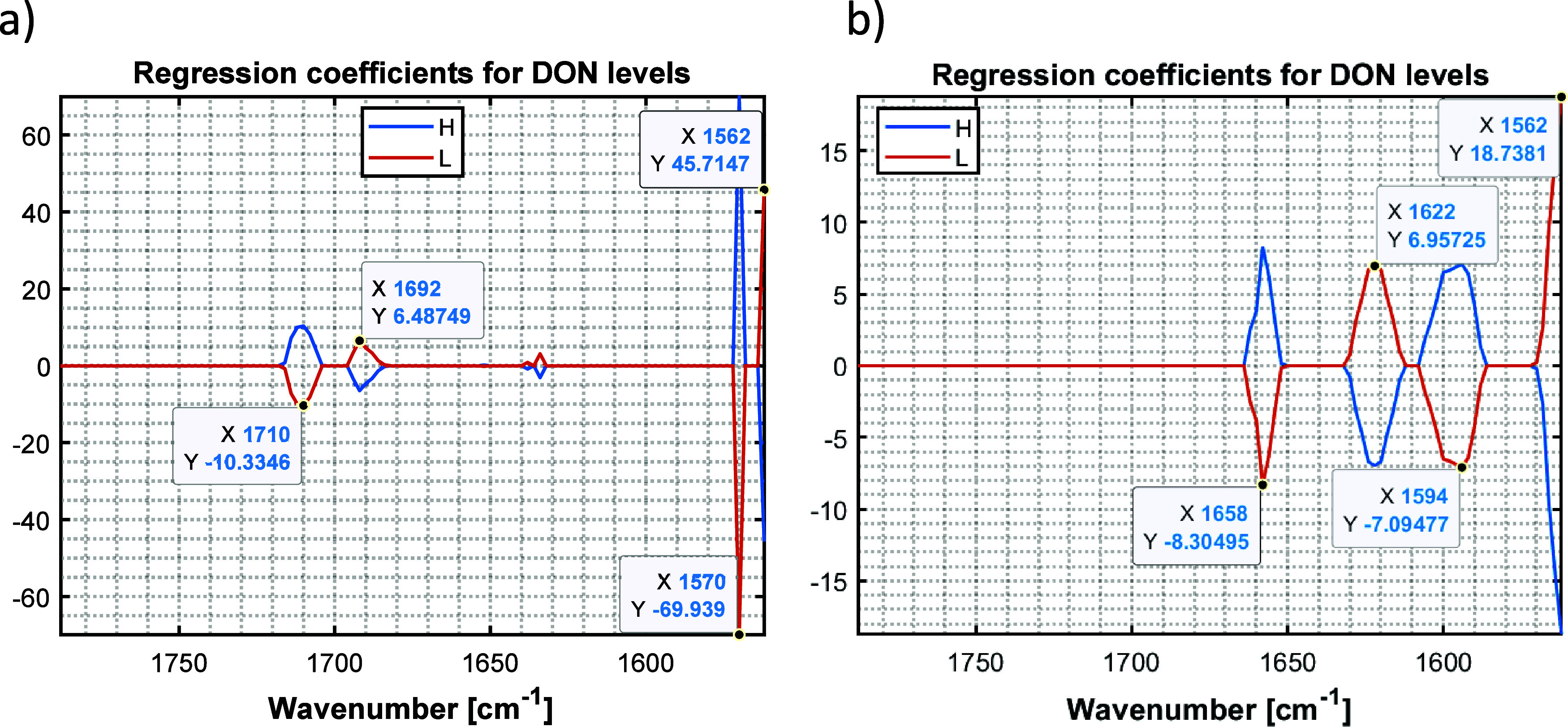
SPLS-DA regression coefficients of the models obtained with the laser IR spectrometer from the different solvents (a) water (100) and (b) ethanol:water (30:70). Blue lines correspond to the high DON contaminated samples (DON > 1250 μg/kg), and orange lines correspond to the low DON contaminated samples (DON < 1250 μg/kg).

Furthermore, the regression coefficients of the models were investigated (Figure 4). This allowed for a detailed interpretation of the models. In both cases, the main contributions come from variables related to proteins (1700–1400 cm^–1^),^37^ which is consistent with the findings obtained by Bellesi et al. and Call et al., demonstrating that the presence of *Fusarium* species influences the gluten composition in wheat.^43,44^ In addition, the wavenumber 1658 cm^–1^ in the ethanol:water (30:70) model aligns with the wavenumber of the band maximum in the IR spectra of the wheat extract (Figure 3), meaning that the changes in wheat matrix caused by fungi growth have strong influences on the model. In turn, one of the wavenumbers (1570 cm^–1^) with a high regression coefficient in the water model is derived from the lipid range within the spectra. It is evident that different information is captured by the models of water (100) and ethanol:water (30:70) extracts. This confirms the hypothesis that solvents of different polarity extract components at different ratios. These findings are consistent with the protocol described by Call et al. investigating wheat protein fractions, whereby for the extraction of various wheat components, different solvents are utilized. For instance, to extract fat, *n*-hexane is used, while proteins such as albumins, globulins, and amylase-trypsin inhibitors are extracted with a 2% aquatic solution of NaCl. The extraction of gliadins is performed with 70% ethanol, while glutens are extracted by solvents mixture.^44^

In summary, the obtained findings demonstrate the capability of the IR laser spectrometer to predict DON contamination in wheat after an extraction step utilizing eco-friendly solvents and emphasize the general potential of QCL-based spectroscopy for confirmative analysis of grains on-site or in a lab as a competitive alternative to the conventional FTIR approach. Despite the achieved classification accuracy, the device still faces certain limitations, including difficulty for untrained users to operate the system and the required safety precautions.

As the next step, model validation via external data sets is required to prove the device transition into real-world scenarios. In the following, the device performance should be verified by DON prediction under conditions relevant for food industries.

### 3.2 IR MEMS Spectrometer for DON Analysis

**Figure 5:**
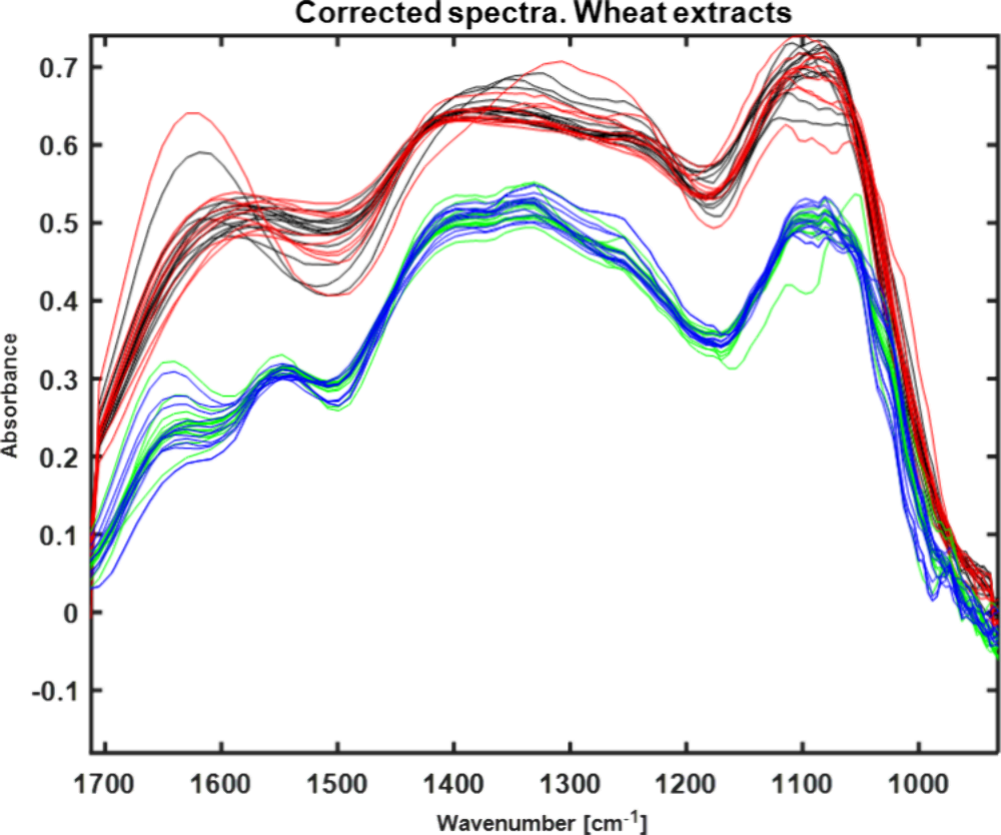
Preprocessed IR spectra of wheat extracts obtained with the IR MEMS spectrometer with water (100) as the extraction solvent (black line–low contaminated sample, red line–high contaminated) and using ethanol:water (30:70) (blue line–low contaminated, green line–high contaminated).

Exemplary IR spectra obtained with an IR MEMS spectrometer are shown in Figure 5. Again, while individual characteristic peaks of components may not be singled out, the spectral pattern is dependent on the extraction solvent, as observed for the IR laser spectrometer in this study and in the research conducted previously with FTIR spectrometer.^29^

The classification performance into high and low contamination has been tested using SPLS-DA models.^28^ The results are presented in Table 4. The cross-validation accuracies for both solvents are inferior compared to the results from the IR laser spectrometer. However, this is anticipated as the device is intended for rapid on-site preliminary screening. Nevertheless, the classification accuracy for water (100) and ethanol:water (30:70) extracts exceeds 80%, which is sufficient for fast mycotoxin screening in the field conditions.^2,45^ The ethanol (30:70) model, which uses 9 latent variables, is more complex than that of the laser device. This complexity enhances its ability to capture the intricate patterns associated with DON contamination. It should be noted that the specificity of the model exceeds 80%, which indicates its ability to predict true negatives. In turn, the number of false negatives for the water model is 1, while for ethanol:water (30:70), it is 2, which is comparable to the numbers gained with the laser device. It is worth noting that although pure water is a more environmentally benign solvent, the ethanol mixture is more appropriate for analysis using this technology due to its significantly shorter evaporation time. Specifically, pure water requires 40 min for complete evaporation, whereas the ethanol (30:70) mixture reduces this duration to 7 min, significantly streamlining the sample preparation process.

**Table 4:** SPLS-DA Classification Results of the High and Low DON-Contaminated Wheat Extracts Obtained via the IR MEMS Spectrometer^a^

**solvent**	**Acc**_**cv**_**, %**	**#LVs**	specificity, %	sensitivity, %	**no. of false negative samples**
ethanol:water (30:70)	84.6%	9	83%	86%	2
water (100)	83.3%	4	70%	93%	1

a Acc_CV_ – accuracy of cross-validation, #LV – number of latent variables.

**Figure 6:**
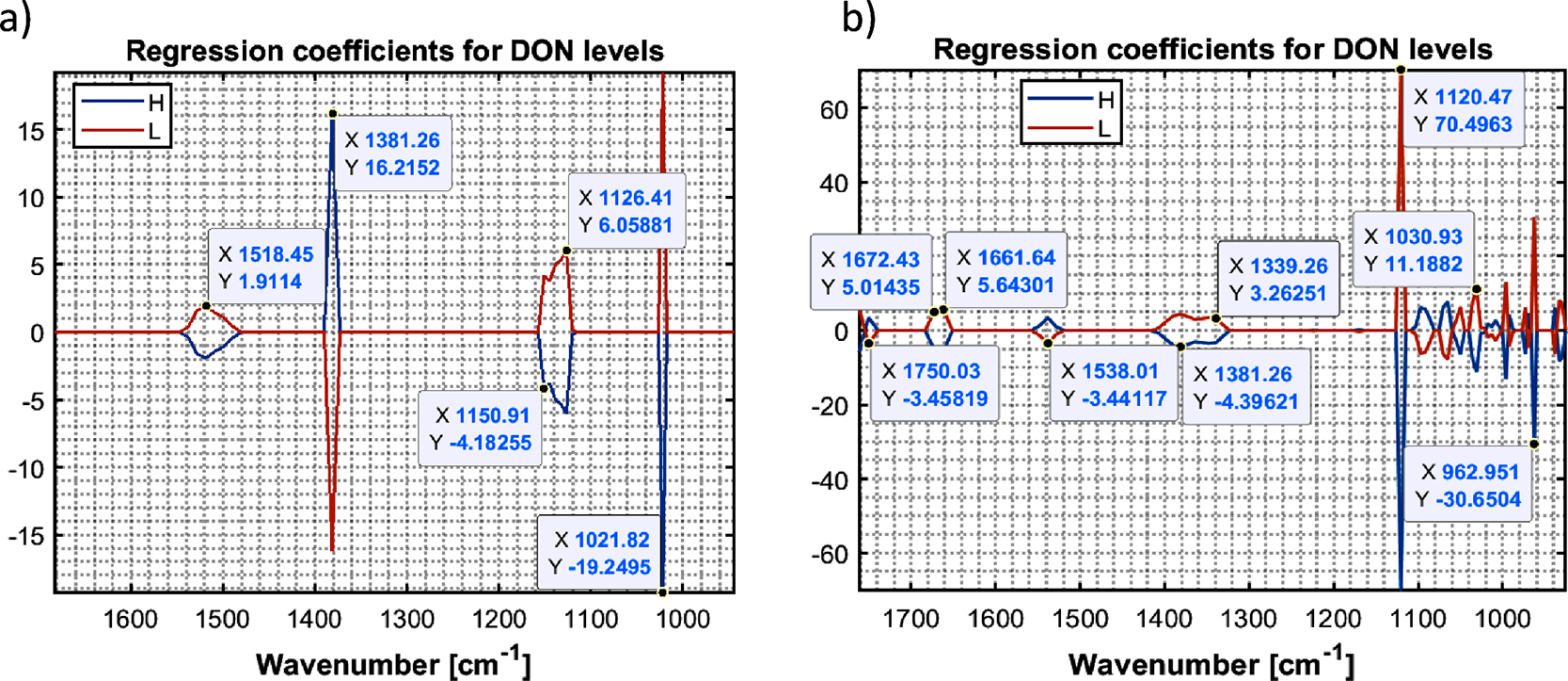
SPLS-DA regression coefficients of the models obtained with the IR MEMS spectrometer from the different solvents (a) water (100) and (b) ethanol:water (30:70). Blue lines correspond to the high DON contaminated samples (DON > 1250 μg/kg), orange lines correspond to the low DON contaminated samples (DON < 1250 μg/kg).

To more deeply understand the models, the regression coefficients were examined. Figure 6 shows that, in contrast to the IR laser spectrometer, it is primarily the carbohydrate typical absorption range (1200–900 cm^–1^)^40^ that contributes to the classification results. This can be explained by the fact that the IR laser spectrometer covers only the spectral range 1850–1550 cm^–1^, while the IR MEMS spectrometer covers a wider range of 1818–909 cm^–1^. This aligns with the findings obtained with the FTIR spectrometer by Fomina et al., whereby for all extraction solvents the major contribution to the models comes from wavelengths in the carbohydrate absorption range.^29^ The obtained results are in line with the research from Bacala et al., which demonstrated that not only proteins are influenced by *Fusarium* species, but also carbohydrates such as starch that are subject to alterations.^46^

The achieved results underline the potential of hand-held IR MEMS spectrometers for on-site preliminary screening of DON in wheat and propose its applicability to different scenarios along the food supply chain, such as harvesting, transportation, and storage offering a compact low-cost solution, while however sacrificing accuracy of prediction in comparison to the FTIR approach. Similar to the laser device, the next development step requires model validation via external data sets and in relevant real-world scenarios (e.g., agricultural organizations or food processing companies).

## Abbreviations

Acc_cv_: accuracy of cross-validation
ATR: attenuated total reflectance
CV: cross-validation
DON: deoxynivalenol
ELISA: enzyme-linked immunosorbent assay
EMSC: extended multiplicative scatter correction
EU: European Union
EC-QCL: external cavity quantum cascade laser
FTIR: Fourier transform infrared spectroscopy
FTIR: Fourier transform mid-infrared spectroscopy
FTNIR: Fourier transform near-infrared spectroscopy
GaAs: gallium arsenide
LC-MS/MS: liquid chromatography-tandem mass spectrometry
LOD: limit of detection
MCT: mercury cadmium telluride
MEMS: microelectromechanical systems
MIR: mid-infrared
MSC: multiplicative scatter correction
NN: neural network
PC-LDA: principal component linear discriminant analysis
PLAD: pyroelectric line array detector
PLS-DA: partial least squares discriminant analysis
PCA: principal component analysis
QCL: quantum cascade laser
SNR: signal-to-noise ratio
SPLS-DA: sparse partial least squares discriminant analysis
SVM: Support Vector Machines
LV: Latent Variable
LVF: Linear Variable Filter
ZnSe: zinc selenide
